# Application of the BP Neural Network Model in the Coordinated Development of Tourism Economic Networks in the Guangdong-Hong Kong-Macao Greater Bay Area

**DOI:** 10.1155/2022/3726696

**Published:** 2022-06-02

**Authors:** Yuguang Ren, Wendong Chen, Xiaolei Chen

**Affiliations:** ^1^School of Economics and Management, Guangzhou College of Commerce, Guangzhou, Guangdong 510555, China; ^2^School of Manage, Guangzhou College of Commerce, Guangzhou, Guangdong 511363, China

## Abstract

In the present era, people are facing enough challenges, either personally or technically, and some people do suffer from having to keep up with the current technological improvements. The Guangdong-Hong Kong-Macao Greater Bay Area (GHKMGBA) is one of the areas that has been highly managed for tourism with economic networks in it. Here, the exploitation results in underground spacing management automate the alleviation process using various methods. Apart from the characterization, it is one such mandatory consideration to create a master plan with the rise of utilization and urban underground spacing planned to be placed among large cities for tourism is considered. This paper focuses on the strategy to understand the concepts and popularisation of urban underground spacing. This matter is followed by a complementary approach with a supportive technical system. This research focuses on economic development in the cities of GHKMGBA, and the analysis is carried out with the implementation of the backpropagation neural network (BPNN).

## 1. Introduction

Government and academic organisations are increasingly concerned about how the bay area is being used as its economic importance develops. The San Francisco bay area is where the term “bay area” was first used. In today's world, there are three bay regions: New York, San Francisco, and Tokyo. For instance, a prominent intersection in New York City's financial centre functions as crossroads for the whole country [1]. Most of the country's population resides in cities, and manufacturing accounts for 30 percent of the GDP. It is becoming increasingly difficult to imagine a future without the bay area's rapid expansion. Foreign media have reported that Chinese construction in the San Francisco bay area began much later than expected. The Chinese bay regions were first proposed in the 1980s by a researcher [2]. The phrase “bay area” was originally used in the Pearl River Delta Township's “Coordinated Development Planning of Pearl River Delta Township Group (2004–2010)” plan. Establishing the NDRC, as well as the departments of foreign affairs and trade, was a major undertaking. The “Greater Bay Area” never existed. A few tips for improving regional cooperation in the Pearl River Delta region were made public by the State Council in March of this year. As a result of Hong Kong and Macau's effect on the Greater Bay Area and researcher economic belt, Guangdong's economy is expected to grow [3]. The Greater Bay Area and the world-class city groups of Shenzhen, Guangzhou, and Macao are growing together with Guangdong. Hong Kong's and Macau's prime ministers have both resigned as of this writing, according to the Hong Kong government's official website [4]. There has been a strong push for urban agglomeration policies in the Greater Bay Area since January. As a result, Hong Kong and Macau were included in the plan for the Greater Bay Area. In addition to Hong Kong and Macau, the Greater Bay Area includes Guangdong. According to historical and current global trends, our region's economy appears to be best suited for tourist development [5]. According to a number of scholars, these tourist attractions make a major contribution to the local economy. Furthermore, Guangdong's GDP is boosted significantly by tourism in terms of relevance, consumption complementarity, and other qualities, according to a quantitative study [6]. According to recent studies, Chinese tourists are increasingly travelling outside of China. In this study, there is no intermediate demand, but rather a high input rate and a weak connection between the two variables. According to the authors, the economic infrastructure of the country is increasingly geared toward tourist destinations. According to new research, the expansion of the Beijing tourism industry has been linked to China's agricultural boom [7]. The tourist and leisure businesses in the greater San Francisco bay area have received scant attention from academics (Guangdong, Hong Kong, and Macao).

The economic impact of Guangdong, Hong Kong, and Macau on the San Francisco bay area is substantial. The area around ports in coastal cities and urban areas is usually referred to as “bay areas.” The marine industry uses the term “port groups” to refer to clusters of seaports and coastal communities [8]. There is a lot of interest in this research because of its proximity to the San Francisco bay area. The inquiry covered nine cities and two SARS outbreaks. People from Guangdong, Hong Kong, and Macau may learn about the bay area's attractions and recreational activities. No statistics have been compiled on the tourist and leisure industries in Guangdong, Hong Kong, or Macau. The tourist and leisure industry is difficult to evaluate because of its enormous breadth and complexity. This investigation focuses on the bay area's tourist and recreation industries. Bay area is a word that incorporates all of the bays and ports in a certain location [9]. New York City, San Francisco, and Tokyo are just a handful of the bay area's most developed cities. Guangdong, Hong Kong, and Macau's tourist companies are important to the region's economy. Coastal tourism is a major and increasing sector in San Francisco and the surrounding areas. In 2013, between 10% and 91% of New York City's total added value was created by the primary and secondary sectors. The marine economic plan of the Busan bay area aims to improve coastal economic growth [10]. As a result, the tourist industry in the Singapore Strait is highly dependent on fluctuations in the exchange rate. The majority of the region's marine economic output is generated by the coastal tourist business in the San Francisco bay area, which has experienced a rise in job opportunities in recent years. Since tourist and leisure businesses are booming in the city, it is swiftly advancing to the top of the local rankings [11]. Guangdong Province is an important marine area since it is situated along vital shipping channels. In the long run, people may say that shipbuilding is Guangdong's area of strength. Maritime tourism increased Zhuhai's GDP by 34% in 2013. Zhuhai is China's easternmost city. (GDP). In 2014, beach tourism in China's maritime business generated revenues of 888.2 billion yuan [12]. In the San Francisco bay area, 85.3 percent of citizens live in Guangdong's coastal tourism business, which needs more attention. It is important to keep in mind a bevy of financial factors. A civic obligation is to plan and maintain public events and landmarks. The development of the tourist and leisure sectors is critical to the long-term prosperity of Hong Kong and Macao [13]. Even without the world-class marine and transportation infrastructure, tourists flock to Hong Kong for its distinct culture and one-of-a-kind experience. With the “free exercise” provision of the “one nation, two systems” policy, some believe that Hong Kong's recent revival as an entertainment and tourist destination may be connected to the policy. In official estimates, Macao is on course to become one of the world's most popular tourist destinations (WTLC). The National Development and Reform Commission of China officially recognised Macao as an amusement and tourist destination in 2008 (NDRC) [14]. The Macao World Tourism and Recreation Center obtained financial assistance from the State Council in November 2010 (government). It was signed in 2011 to promote the building of an international tourism destination of world-class status and assure adequately varied economic growth between Guangdong and Macao. Through Macao, they hope to exert more influence on the country's long-term development. This practise will be allowed to continue even in the Greater Bay Area China World Tourism and Leisure Center.

Machine learning can make predictions based on a wide range of data. Reliable predictions are critical to the tourism industry's long-term sustainability. Smartphone recommendation systems can be used to improve the tourism industry's marketing and planning [15]. The field of computer science known as “machine learning” focuses on teaching computers to learn from the data they are fed [16]. The phrase “machine learning” encompasses a wide range of subfields. Other methods of reinforcing behaviour include the use of incentives. Machine learning, a branch of artificial intelligence, employs algorithms to sort through enormous datasets. The process of turning raw data into information is an effective way to broaden one's horizons and get fresh insights. These goods are extremely important to the tourism industry. Machine learning is heavily dependent on statistical data in the tourism industry. The number of visitors visiting a given country or region may be seen on several websites (UNWTO) [17]. Visitor arrivals are included in every year's World Visitors Organisation report on tourism statistics. These data may be used in a variety of ways to forecast future visitor demands. Photos and maps from the Internet are frequently used in the development of tourism-related machine learning. More and more people are relying on visual aids like photos and videos to help them better comprehend a new environment. An image-based deep learning model on computers may be used to analyse travellers' behaviour and thoughts. Geotagging is a popular way for tourists to document their travels and the places they have visited [18]. The travel and tourist industry relies heavily on maps as a source of information. There is no tourist map that does not include Google Maps. It is not uncommon for text to be the primary source of data for machine learning algorithms. People can learn a lot about the tourism industry by reading a book. In the late 1950s and early 1960s, researchers used the phrase “machine learning” to describe the progress of artificial intelligence. Many travellers use geo tagging to keep track of where they have been and what they have seen while on the road. The travel business relies on maps as a kind of data. Text is the primary data source for machine learning in the tourism business [20]. Using text to acquire tourist information is a valuable strategy. This study focused on analysing the tourist attractions in the Guangdong-Hong Kong-Macao Greater Bay Area using the BP neural network.

### 1.1. Motivation for the Study

A well-managed tourist destination with strong economic ties is the Guangdong-Hong Kong-Macao Greater Bay Area (GHKMGBA). Here, the exploitation results in the management of subsurface space, and the alleviation process is automated in many ways. A master plan must be drawn up in light of the rise in usage and urban subterranean spacing planned among significant cities for tourism, as well as the categorization. The BP neural network is the synchronized development of the tourism economic networks in the Guangdong-Hong Kong-Macao Greater Bay Area which is considered in this research. Processing elements are represented as x, which appears to be the analysis value of physical quality education. The western surface nodes and the output nodes appear to be the analysis value of the quality of the physical education. Because the input BP neural network model in the coordinated development of tourism economic networks in the Guangdong-Hong Kong-Macao Greater Bay Area node equals the input. The midlayer base station's output information is a significant contribution. The activation function only has one base station, which also accepts input from a middle layer node and outputs the teaching standard evaluation effects.

## 2. Materials and Methods

According to the statement, knowledge within the demographic and economic literature on urbanization has been frequently used in the indication methods. For example, this urbanization technique helps in the development of indicators and there is enough intricate contact in the development process, which is a kind of state of the population. However, during the year 2009, there was a report from the state of population analysis that showed that most developed countries had been considered preurbanized countries. Moreover, the remaining countries being developed with the least methods result in lower urbanization. Therefore, the term “urbanization” is being used as a proxy variable that is a resonating proxy variable for development. Furthermore, development has the impact of more significant connotations and is familiar with the reports taken from human developmental analysis. This term has been explained with several terms of longevity, life expectancy, literacy, and other income rate management.

**Figure 1:**
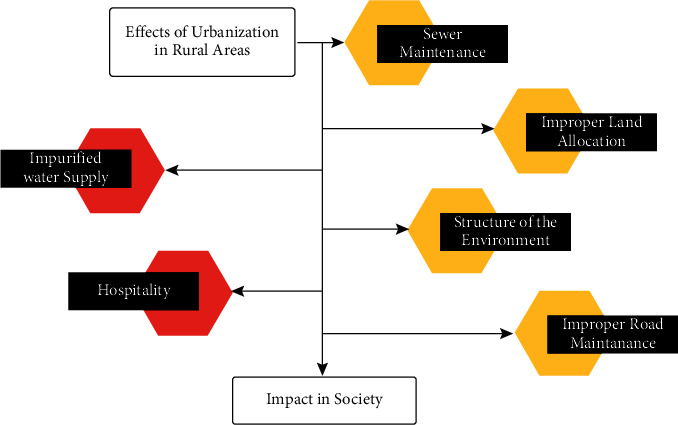
Graphical form of the living ecological space.

Usually, the areas used to connect the bays, harbours, and any other islands located to the nearer location is the actual meaning of the bay area. In recent days, the top urban agglomeration is mainly developing with the coastal bay areas like Tokyo and the New York bay area. Li is a great expert and believes that the rise of bay area economy coverage is the sum of 5 conditions that create a strong and natural industrial-based cluster and the second method is to overtake the broad economic hinterland. Finally, it also deals with geographical conditions. However, all these methods cannot be imagined under the demographics method because the reports are taken from a popular article that is a review-based development for an Indian journal. Both the cases of urbanization and the indicator of development do come under demographic transition and few migration systems. If there is a country that is urbanized and does not get much popularity as other countries, then this will not be considered by the experts in knowledge. While listing out the possibilities, it would reach the massive path that relates permutation and other possibilities. We cannot imagine a world untied with enough support, which does not consist of any urbanization process. According to economic growth, people do suffer without knowing about urbanization. The graphical representation formulates the entire data method in a clear method, which helps to have an overview of two entire processes. With an example of novel literature, it is said that under the future land simulation model, the method is being integrated with the evaluation of various environmental resource management and that suits the sustainable prediction process is shown in Figure 1.

BP neural network model in the coordinated development of tourism Economic Networks in the Guangdong-Hong Kong-Macao Greater Bay Area has the input data node.

We input data node *m*_*i*_, *i*={1,2,…, *x*}, where *x* reflects teaching quality evaluation. The input to the attribute ∑_*j*=1_^*H*^(*H*_*j*_^−1^ − 1)^2^aids in the coordination of tourism economic network development in the Guangdong-Hong Kong-Macao greater bay area. Finding H, the middle of the sampling node is represented in (1).

(1) $$\begin{array}{c} H_{j}=\sum_{j=1}^{x}\frac{1}{\left[{\left(H_{j}^{-1}-1\right)}^{2}\right]}+\sum_{j=1}^{x}\varphi_{ij}m_{i}\times \sum_{j=1}^{H}{\left(H_{j}^{-1}-1\right)}^{2}. \end{array}$$

The outcome is the coordinated development of tourism area economic networks as in the following equation:

(2) $$\begin{array}{c} R_{j}=\sum_{j=1}^{x}\frac{1}{{\left\{1+{\left[{\left(\sum_{i=1}^{x}\varphi_{j}R_{j}\right)}^{-1}-1\right]}^{2}\right\}}^{\prime }}+\sum_{j=1}^{x}\frac{1}{\left[{\left(H_{j}^{-1}-1\right)}^{2}\right]}. \end{array}$$

Here, *φ*_*j*_ signifies the strength from such an input datatype access point *H*_*j*_^−1^ to a frame node's centre *j* and *R*_*j*_ denotes its graph's factor, its *i*^th^ teaching quality evaluation index.

Node of activation function: there are only *S* nodes with in the destination node as shown in the following equation, and the data are the bandwidth of the frame node's centre:

(3) $$\begin{array}{c} S=\sum_{j=1}^{x}\frac{1}{{\left\{1+{\left[{\left(\sum_{i=1}^{r}\varphi_{j}R_{j}\right)}^{-1}-1\right]}^{2}\right\}}^{2^{\prime }}}+\sum \left\{1+{\left[{\left(\sum_{i=1}^{x}\varphi_{j}R_{j}\right)}^{-1}-1\right]}^{2}\right\}. \end{array}$$

The learning optimization strategy is defined as the overall average of all such sum of squares sum ∑_*i*=1_^*x*^*φ*_*j*_*R*_*j*_. the error between the overall performance and the measurement value of represented as G, and the results are derived with equation (4)

(4) $$\begin{array}{c} G=\sum_{i=1}^{x}\varphi_{j}R_{j}+\left(\frac{1}{M}\right)\sum_{m=1}^{m}{\left[\bar{s}-s\right]}^{2}=\left(\frac{1}{M}\right)\sum_{m=1}^{m}G_{j}. \end{array}$$

Despite the BP neural network evaluating process's shift in the *δG* framework, the goal of network education appears to minimize *τ* by modifying the network's login procedure. The training algorithm technique *δc*_*ij*_ is used to change the delinking as the following equation:

(5) $$\begin{array}{c} G_{ij}=\sum \left\{\begin{array}{c} \varphi_{ij}=-\tau \left(\frac{\delta G}{\delta c_{ij}}\right),\\ \varphi_{j}=-\tau \left(\frac{\delta G}{\delta \varphi_{ij}}\right). \end{array}\right. \end{array}$$

Furthermore, the coordinated development of tourism economic networks is found in the area with such a rate of learning. The *d*_*i*_*φ*_*j*_*R*_*j*_^2^ quantity of communication optimization parameters between the source data network and the midlevel access point is therefore shown in the following equation:

(6) $$\begin{array}{c} \varphi_{ij}=d_{i}\varphi_{j}R_{j}^{2}\left[1-\sum_{i=1}^{x}\varphi_{ij}d_{j}\right]\omega_{j}. \end{array}$$

The number of connection area optimization parameters is tourism economic networks as follows:

(7) $$\begin{array}{c} \varphi_{j}=\sum_{j=1}^{r}s^{2}R_{j}\left[1-\sum_{j=1}^{r}\varphi_{j}R_{j}\right]{\left[\bar{s}-s\right]}^{2}. \end{array}$$

So, using this framework, the neural network's communication weight may be defined to apply the ∑_*j*=1_^*r*^*φ*_*j*_*R*_*j*_ optimization technique of a specific neural network and the ${\left[\bar{s}-s\right]}^{2}$ coordinated development of tourism economic networks in the *s*^2^*R*_*j*_ area in accuracy among the total performance with different sampling values can be decreased. The system will be in a higher optimization phase, as shown in the following equation by employing an optimised neural network:

(8) $$\begin{array}{c} \min\left(G\right)=\sum_{j=1}^{r}\int \left(\varphi_{1},\ldots ,\varphi_{x}\right), \end{array}$$

where *φ*_1_ is the as-a-whole inaccuracy of network training and *φ*_1_,…, *φ*_*x*_ are the continuous weights now since the strong and united number system includes the weights of the network of input that coordinated the development of tourism in the context of economic networks. This process is performed with through the area data access points along with centre layer endpoints. The model parameters are categorized as concentrators and output node modules, with n as the number of network parameters. Among several parameters, $\bar{s}$ and *s* are *φ*_1_ variables that denote the lowest and maximum values of change.

The coordinated development of tourism economic networks in the process of analytics using the BP neural network algorithm appears to be a low capacity problem. Because a person's areas of interest are essentially equivalent to ∫(*φ*_1_,…, *φ*_*x*_) ability, the description of an optimization process has a significant impact on the BP neural network algorithms. Because of the close navigation link between the *G* < *U* optimization process and the BP neural network method, the GU strength training computational strategy shown in the following equation is used:

(9) $$\begin{array}{c} \int_{i}\sum_{i=1}\left\{\begin{array}{cc} U-G, & G<U,\\ 0, & G\geq U, \end{array}\right.+2\left(\frac{\int_{1}U-G,G<U}{U}\right). \end{array}$$

In equation (9), *e* represents the training optimization issue and *G* represents the sum of all *G* ≥ *U* in the current generation.

The guidelines of coordinated development of tourism economic networks in the area given as follows are utilised for the *M*_*r*_ classification. Classification of the parameters in ∫_1_(*U* − *G*)/*U* ∈ [0,0.5] provides the efficiency of convergence and to prevent redundant integration caused by efficient gene declassification.

(10) $$\begin{array}{c} M_{r}=\sum \begin{array}{cc} \left(\frac{\int_{1}U-G,G<U}{U}\right), & \frac{\int_{1}\left(U-G\right)}{U}\in \left[0,0.5\right],\\ \left[1-2\left(1-{\left(\frac{\int_{1}U-G,G<U}{U}\right)}^{2}\right)\right], & \frac{\int_{1}\left(U-G\right)}{U}\in \left[0.5,1\right]. \end{array} \end{array}$$

Its min and max technique is employed for normalising handling ∫_1_(*U* − *G*)/*U* ∈ [0.5, 1]. This implementation is a viable implementation for information processing that can effectively keep its own original definition to avoid data redundancy. The normalization function is represented in the Equation (10) to utilize in this article for such input information is as follows: The process of tourism economic networks in the area compressing a wide range of information into the scope [0, 1] is known as standardization ∫_1_(*U* − *G*)/*U* ∈ [0.5, 1]. The solution for the economic network is given in following equation:

(11) $$\begin{array}{c} d^{\prime }=\sum_{i=1}^{x}\frac{d-d_{\min}}{d_{\max}-d_{\min}}+\sum_{U}^{G}\left(\frac{\int_{1}U-G,G<U}{U}\right). \end{array}$$

The standardization process entails converting the dataset's small and large outlier information into a normal random variable with an overall average value of 0 and a confidence interval of 1. The following equation represents the same:

(12) $$\begin{array}{c} d^{\prime }=\sum_{i=1}^{x}\frac{d-{\bar{d}}_{\min}}{\sigma }+1-\sum {\left(\frac{\int_{1}U-G,G<U}{U}\right)}^{2}. \end{array}$$

Each data centre is composed of three layers: input layers, hidden layers, and convolution layers, with the weight of each layer being *β*, *γ*, and *α*. It indicates that specific *φ*_*y*′*y*_*q*_*y*′_^*t*−1^ documentation is kept in the neural network receptors after each cycle of data transmission. It must enter a *φ*_*y*_(*q*_*y*′_^*t*−1^) next nerve cell as new information and impact the subsequent data output. Equations (13), (14), and (15) represent the calculation for corresponding input nodes, the original input of the hidden units, and the output layer in the coordinated development of tourism economic networks in the area at the given time step t.

(13) $$\begin{array}{c} \beta_{y}^{t}=\frac{\int_{1}\left(U-G\right)}{U}\in \left[0,0.5\right]\times \sum_{i=1}^{M}\varphi_{iy}d_{i}^{t}+\sum_{y^{\prime }}^{M}\varphi_{y^{\prime }y}q_{y^{\prime }}^{t-1}, \end{array}$$

(14) $$\begin{array}{c} \alpha_{y^{\prime }}^{t-1}=\frac{\int_{1}\left(U-G\right)}{U}\in \left[0.5,1\right]\times \varphi_{y}\left(q_{y^{\prime }}^{t-1}\right), \end{array}$$

(15) $$\begin{array}{c} \gamma_{y^{\prime }}^{t-1}=2\frac{\int_{1}U-G,G<U}{U}\times \sum_{y=1}^{M}\varphi_{y0}q_{y}^{t}. \end{array}$$

## 3. Results and Discussion

**Figure 2:**
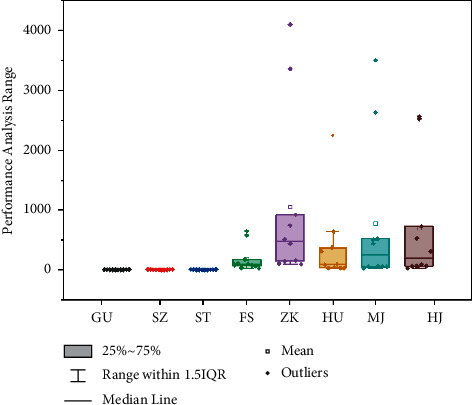
Performance analysis of the BP neural network model in the coordinated development of the tourism economic networks.

Export records are commonly utilised as a reliable measure of economic productivity when evaluating the economic growth within city groupings. Guangdong's government is providing city-level statistics from many manufacturing industries; the BP neural network model is used in the coordinated development of tourism economic networks in the Guangdong-Hong Kong-Macao Greater Bay Area. We input data node *m*_*i*_, *i*={1,2,…, *x*}, where *x* reflects teaching quality evaluation. The input to the ∑_*j*=1_^*H*^(*H*_*j*_^−1^ − 1)^2^ coordinated development of tourism economic networks in the Guangdong-Hong Kong-Macao Greater Bay Area where *H* is the middle of the specimen node is represented and determined in Figure 2. These numbers are utilised to replace the export of different city ranges of economy by examining the characteristics of Chinese cities when assessing their economic difficulty. This study computes the disclosed competitive advantage for every manufacturing industry of the cities just at the municipal level and then analyses the cities' versality and variety to reflect their major impact on economic complexities.

**Figure 3:**
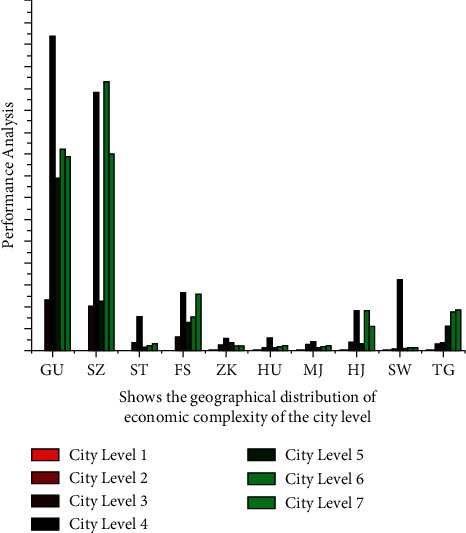
The geographical distribution of the tourism economic complexity of the network in the city level of the Guangdong-Hong Kong-Macao Greater Bay Area.

The Harvard University-developed economic complexity framework has various applications in assessing the competencies of economies at regional level. Financial complexity is built upon the analysis of such an economy's variety and ubiquity and also acquired to disclose the relative advantage in distinct industrial sectors to represent the information barrier of the manufacturing industry for possessing the economic standard in the tourist area. The neural network communication weight may be defined to apply the ∑_*j*=1_^*r*^*φ*_*j*_*R*_*j*_ optimization technique of a specific neural network, and the ${\left[\bar{s}-s\right]}^{2}$ coordinated development of tourism economic networks in which *s*^2^*R*_*j*_ represents the area chosen for analysis in terms of accuracy. The difference between these total performances with different sampling values can be decreased and the results are given in Figure 3. One of the two complimentary measures for intellectual assets is ubiquity, which evaluates how many items an economy produces. And ubiquity, which measures the export level of various countries through trending technologies.  Table 1 examines normalization to ensure that tiny economies and industrial sectors are treated equitably.

**Figure 4:**
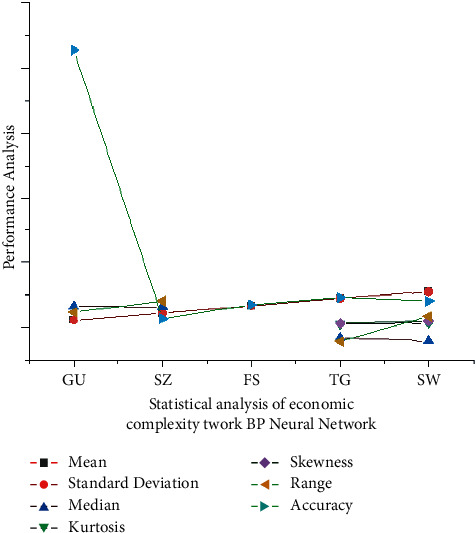
Statistical analysis of the tourism economic complexity using the BP neural network model.

Hong Kong seems to have the greatest average economic sophistication, followed by Guangzhou, Shenzhen, and then Zhuhai. Among cities also with lowest mean quantitative methodology are Huizhou, Chaozhou, Yunfu, and Heyuan, as well as Jiangmen. Here, *φ*_1_ is the as-a-whole inaccuracy of network training, and *φ*_1_,…, *φ*_*x*_ are the continuous weights now since the strong and united number system includes the Weights of the network input provide the coordinated development of tourism economic networks, and the results are given in Figure 4. Among them, $\bar{s}$ and $\bar{s}$ are *φ*_1_ variables that denote the lowest and maximum values of change. Shenzhen, Zhuhai, Foshan, Zhongshan, and Yangjiang now have largest standard deviation in terms of financial complexity during the last 16 years, while Huizhou, Shantou, Heyuan, Jieyang, and Jiangmen have the minimum. The statistical report analysis of tourism economic complexity using the BP neural network model in different cities in the Guangdong-Hong Kong-Macao Greater Bay Area is shown in Table 2.

**Figure 5:**
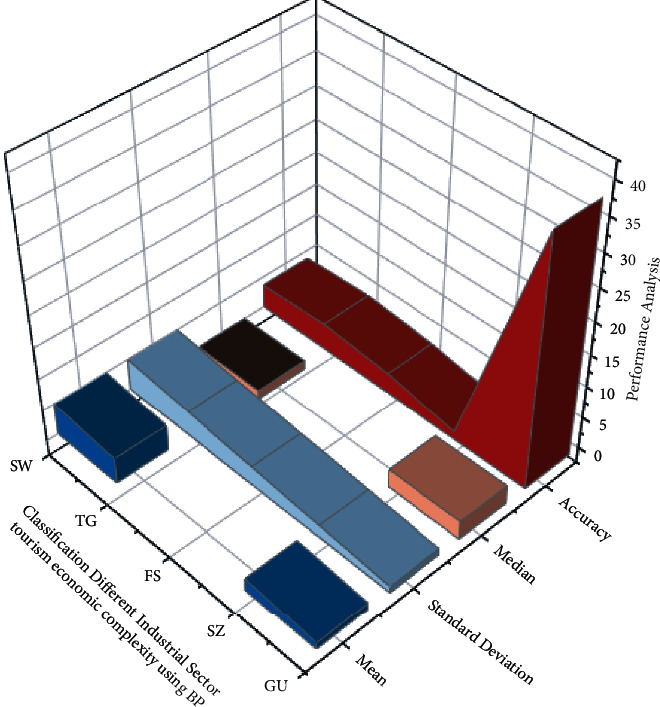
The classification of tourism economic complexity of different industrial sectors in Guangdong-Hong Kong-Macao Greater Bay Area using the BP neural network model.

Due to China's tremendous economic expansion over the previous two decades, its academic community has paid close attention to the effects within the Chinese market. The southern area of China is the wealthiest of the country's economic groupings. The classification of different industrial sector tourism economic complexities in the Guangdong-Hong Kong-Macao Greater Bay Area using the BP neural network model is shown in Figure 5. It is critical to perform an in-depth investigation to identify the region's economic viability. The economic complexity model has been used in 5 main cities, as in the Guangdong-Hong Kong-Macao Bay Area with significantly larger grouping. The research is divided into seven industry sectors. The area process of analytics using the BP neural network algorithm appears to have a low-capacity problem. Because a person's areas of interest are essentially equivalent to ∫(*φ*_1_,…, *φ*_*x*_) ability, the description of an optimization process has a significant impact on BP neural network algorithms. Because of the close navigation link between the variables are represented as *G* < *U*, the optimization process and the BP neural network method discloses the relative advantage of various product sectors, commonalities in product industry specialization through variety of economic makeup, and correlation with geographic area exploration.

## 4. Conclusions

People in the modern period have so many difficulties, both personally and technologically, that it can be taxing for some to keep up with the rapid pace of change. On the other hand, urbanisation and the expansion of underground space have improved in the twenty-first century. China's city of Shenzhen is one of the world's most recognisable bay areas and it would be less if people were making estimates throughout the world. The GHMGB is a well-managed tourist destination with strong commercial ties. Underground space management and many kinds of alleviation are achieved through this exploitation. One such aspect, aside from characterising the area, is the growing demand for urban underground space between major cities, which necessitates the development of a master plan. This study proposed the backpropagation neural network model for analysing the tourist attractions in the desired area.

## Figures and Tables

**Table 1:** Result analysis of the tourism economic complexity of the network at the city level.

City	City level 1	City level 2	City level 3	City level 4	City level 5	City level 6	City level 7
Guangzhou (GU)	326.83	6185.66	651.16	4099.96	2255.15	2628.76	2529.47
Shenzhen (SZ)	7.66	7742.93	579.78	3361.14	640.85	3501.52	2564.45
Shantou (ST)	86.74	977.28	96.55	441.48	52.43	60.92	92.99
Foushan (FS)	126.47	5675.16	176.66	743.22	370.42	441.77	728.98
Shaoguan (ZK)	141.63	460.335	78.68	146.13	94.15	60.68	65.67
Heyuan (HU)	97.05	434.69	36.86	158.06	32.53	53.85	67.36
Meizhou (MJ)	178.47	387.96	68.49	107.73	35.47	52.52	62.27
Huizhou (HJ)	141.52	2624.45	112.45	511.43	89.74	521.34	313.77
Shanwei (SW)	128.07	420.27	26.78	923.11	28.98	30.65	31.75
Dongguan (TG)	27.09	3840.39	85.80	95.36	315.95	501.32	529.24

**Table 2:** Result analysis for statistical of tourism economic complexity using the BP neural network model.

City	Mean	Standard deviation	Median	Kurtosis	Skewness	Range	Accuracy
GU	GU	2.76	0.19	2.93	−2.27	−5.25	2.12
SZ	SZ	2.24	0.34	2.87	−2.24	−1.75	3.66
FS	FS	−1.44	0.26	−1.6	0.19	0.19	−1.87
TG	TG	−2.45	0.78	−1.4	0.45	0.56	−1.90
SW	SW	−1.34	0.95	−1.8	0.65	0.95	1.57

## Data Availability

The data used to support the ﬁndings of this study are available from the corresponding author upon request.
